# Association between the dietary index for gut microbiota and metabolic syndrome in adults: the mediating role of body mass index

**DOI:** 10.3389/fnut.2025.1598664

**Published:** 2025-07-16

**Authors:** Hui Liu, Xin Xu, Zhicui Yao, Jialu Kang, Yongqing Shen, Wei Liu

**Affiliations:** ^1^Faculty of Nursing, Hebei University of Chinese Medicine, Shijiazhuang, China; ^2^Hebei Provincial People′s Hospital, Shijiazhuang, China

**Keywords:** MetS, DI-GM, NHANES, gut microbiota, dietary index, mediation analysis

## Abstract

**Background:**

Dietary patterns influence the onset of metabolic syndrome (MetS) through the modulation of intestinal microbiota. Nevertheless, the relationship between the dietary index for gut microbiota (DI-GM), a novel metric for evaluating the link between diet and microbiota well-being, and its correlation with MetS, as well as the potential mediating role of body mass index (BMI), remains unclear.

**Methods:**

This study examined information from 21,100 individuals participating in the National Health and Nutrition Examination Survey (NHANES) conducted between 2007 and 2020. The association of DI-GM with MetS was assessed using a weighted multivariate logistic regression model, and restricted cubic spline curves (RCS), subgroup analyses, and mediation analyses were performed.

**Results:**

A significant inverse correlation was observed between DI-GM score and the prevalence of MetS. The prevalence of MetS decreased by 8% (OR = 0.92, 95% CI: 0.89–0.95) for each unit of DI-GM. The prevalence of MetS was reduced by 26% in Q4 compared with Q1 (OR = 0.74, 95% CI: 0.63–0.87). RCS analysis further revealed a linear relationship between DI-GM and MetS prevalence. Subgroup analysis showed that the negative association between DI-GM and MetS was more significant in the exercise, non-smoking, and non-drinking population. Furthermore, BMI played a significant mediating role in the association, accounting for 52.71%.

**Conclusion:**

A notable negative correlation exists between DI-GM score and the prevalence of MetS. The promotion of a healthy lifestyle can strengthen this correlation, with BMI serving as a crucial mediating factor. This underscores the potential of dietary interventions that focus on gut microbiota in conjunction with weight management as targeted strategies for the prevention and management of MetS.

## Introduction

Metabolic syndrome (MetS) represents a constellation of metabolic abnormalities including insulin resistance, abdominal obesity, elevated blood glucose, lipid abnormalities, and high blood pressure, posing a substantial worldwide public health concern (1). Research suggests that roughly 25% of the world’s population—exceeding 1 billion individuals—suffers from this condition, while in the United States, approximately one-third of adults meet diagnostic criteria (2). Beyond being a clinical condition resulting from the convergence of multiple metabolic risk factors, MetS significantly increases vulnerability to numerous chronic conditions such as heart disease and type 2 diabetes (3). Consequently, prevention and management of MetS are essential to interrupt the onset of associated diseases, slow disease progression, and reduce the overall risk of morbidity and mortality.

Modifiable lifestyle factors, particularly dietary habits, play a pivotal role in the pathogenesis of MetS. Recent findings underscore their reciprocal relationship with gut microbiota in modulating metabolic well-being. Western dietary patterns, characterized by high fat consumption, disrupt gut microbiota composition by diminishing beneficial bacteria (e.g., Bifidobacterium) and fostering the growth of opportunistic pathogens, thereby reducing short-chain fatty acids (SCFAs) production, compromising gut barrier function, and triggering systemic inflammation that exacerbates insulin resistance and metabolic dysfunction (4). In contrast, diets rich in dietary fiber and polyphenols enhance microbial diversity, promote SCFAs synthesis, and inhibit endotoxin-producing bacteria, thereby collectively improving glucose regulation and lipid metabolism (5, 6). Therefore, gut microbiota is a key mediator linking dietary patterns with MetS development. Traditional dietary indices like the Mediterranean diet (MD) and the Dietary Approaches to Stop Hypertension (DASH) have demonstrated protective relationships with MetS by emphasizing anti-inflammatory and cardiometabolic advantages (7, 8). However, these indices do not explicitly account for diet-microbiome interactions, thereby limiting their capacity to evaluate the microbial mechanisms of dietary impacts. To fill this gap, the dietary index for gut microbiota (DI-GM) was developed by Kase et al. as a novel tool to quantify dietary quality related to gut microbiota health (9). The DI-GM comprises 14 food components: beneficial components (e.g., whole grains, fermented dairy products, soybeans, green tea) that enhance microbial diversity and the production of SCFAs, and harmful components (e.g., red meat, refined grains, high-fat diets) that are linked to microbiota imbalances. Initial research indicates that higher DI-GM scores are linked to a decreased risk of diabetes and depression (10, 11), underscoring their potential as indicators of microbial-mediated metabolic health. Despite advancements, comprehensive epidemiological evidence establishing a connection between DI-GM and MetS is lacking, and it remains uncertain whether body mass index (BMI), a fundamental measure of obesity, mediates the impact of DI-GM on MetS.

Therefore, this study utilized data from the National Health and Nutrition Examination Survey (NHANES) 2007–2020 to investigate the association between DI-GM score and MetS prevalence in American adults, as well as the mediating role of BMI. These analyses aim to provide evidence for developing targeted dietary intervention strategies to address MetS.

## Materials and methods

### Data sources

**Figure 1:**
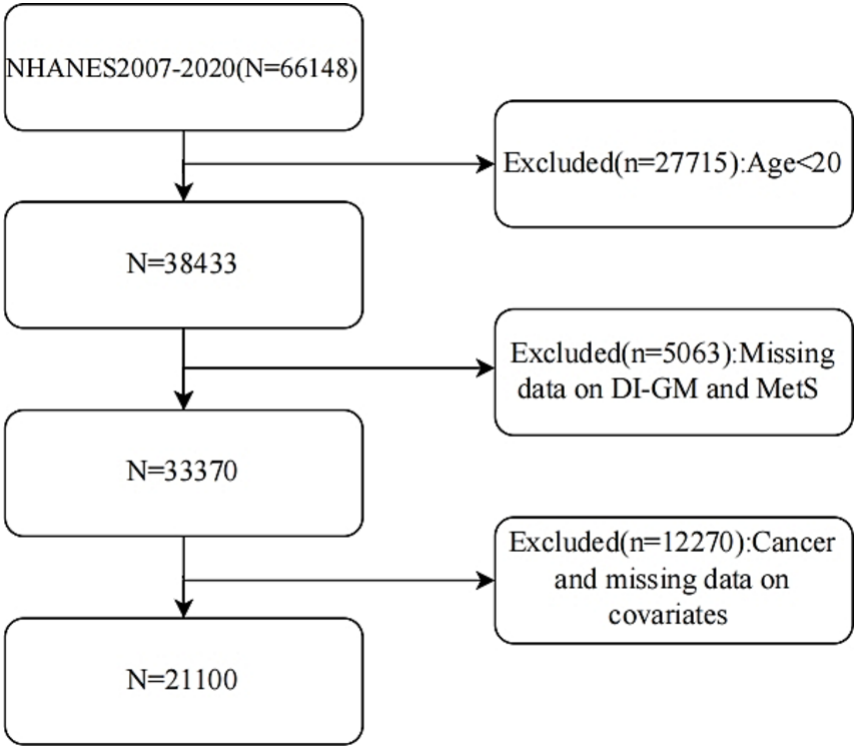
The flow chart of this study.

Conducted by the US National Center for Health Statistics, NHANES represents an ongoing, nationally representative cross-sectional survey that employs a stratified, multistage probability sampling approach. The research protocol received review and approval from the NCHS Research Ethics Review Board, with all participants giving written informed consent. Complete data sets can be accessed directly through the NHANES official website. In this study, we investigated 66,148 participants from 2007 to 2020. Exclusions were made for those under 20, with missing DI-GM or MetS data, a cancer diagnosis, or lacking covariate data. Ultimately, 21,100 participants were included based on strict criteria. The specific inclusion process is shown in Figure 1.

### Dietary index for gut microbiota

To calculate DI-GM, this research examined 24-h dietary recall data from the NHANES database. Participants in NHANES completed two detailed 24-h dietary recall assessments. The initial interview took place at a mobile examination center (MEC), while the second was administered by phone, both documenting participants’ food consumption during the preceding 24-h period. To improve measurement accuracy, we averaged two independent 24-h dietary recall interviews for each participant. The DI-GM scoring system was built based on standards proposed by Kase et al., comprising 14 dietary components: beneficial ones like fermented dairy, chickpeas, soybeans, whole grains, fiber, cranberries, avocados, broccoli, coffee, and green tea; and adverse ones including red meat, processed meat, refined grains, and high-fat diets (with ≥40% of energy from fat) (9). When assessing the link between individual diet and gut health, the DI-GM scoring system used sex-specific medians as the key cutoff. Beneficial components were scored as follows: participants received a score of 1 if their intake met or exceeded the sex-specific median, otherwise they scored 0. For adverse components, scoring was inverted: participants received a score of 0 if their intake was at or above the median (or if fat intake contributed more than 40% of total energy), otherwise they received a score of 1. The total score, summing each component, ranges from 0 to 14.

### MetS diagnostics

Based on the National Cholesterol Education Program’s Adult Treatment Panel III (NCEP-ATP III) criteria (12), metabolic syndrome is characterized by having three or more of these factors: abdominal girth of 102 cm or more in men or 88 cm or more in women; fasting plasma glucose of 100 mg/dL or higher or taking glucose-lowering medication; BP of 130/85 mmHg or higher or using blood pressure-lowering medication; TG levels of 150 mg/dL or higher or receiving medical therapy; high-density lipoprotein cholesterol below 40 mg/dL in men or below 50 mg/dL in women, or being treated with medication.

### Covariates

This study’s covariates comprised demographic and lifestyle factors including age, gender, race, poverty-to-income ratio (PIR), education level (high school education or less, more than high school), marital status (never married, divorced/widowed/separated, married/cohabiting), smoking (yes/no), drinking (yes/no), exercise and body mass index (BMI, weight divided by height squared). Exercise was categorized as participating in a minimum of 10 consecutive minutes of moderate-intensity exercise, physical fitness activities, or recreational activities per week. Data were collected via standardized questionnaires and interview procedures administered by NHANES.

### Statistical analysis

Considering NHANES’ complex stratified probability sampling design, sample weights were incorporated into the analysis. In the descriptive analysis of participants’ baseline characteristics, continuous variables are presented as weighted means ± standard deviations (SD), while categorical variables are reported as actual frequencies and weighted percentages. To investigate the association between DI-GM (both as continuous and categorical variables) and metabolic syndrome, weighted multivariable logistic regression models were used, with results presented as odds ratios (OR) and 95% confidence intervals (CI). Three distinct models were developed: Model 1, which did not account for any covariates; Model 2, which adjusted for age, gender, and race; and Model 3, which further incorporated adjustments for socioeconomic factors (PIR, education level), lifestyle factors (smoking, drinking, exercise), and marital status to mitigate potential confounding influences. The relationship between DI-GM and MetS was assessed utilizing a restricted cubic spline curve (RCS) with three nodes positioned at the 25th, 50th, and 75th percentiles to explore potential nonlinear associations. Subgroup analyses based on sex, age (<60, ≥60), race, exercise, smoking, and drinking were conducted to test for differences and potential variants between different subgroups and tested for interactions between them, but did not perform multiple comparisons. In addition, the Bootstrap method (1,000 replicates) was used to test the mediating effect of BMI, and the average causal mediating effect (ACME, reflecting the mediating effect size), direct effect (ADE, direct effect of DI-GM on MetS), and mediating effect proportion were calculated. Statistical analyses were performed using R version 4.2.0 and DecisionLinnc 1.0, with statistical significance set at *p* < 0.05.

## Results

### Baseline characterization

The research encompassed 21,100 participants, corresponding to a weighted total of 160,008,955 individuals, categorized into four quartiles (Q1-Q4) according to DI-GM scores. Findings demonstrated a meaningful relationship between DI-GM and metabolic syndrome prevalence (*p* < 0.001, Table 1). MetS prevalence decreased from 24.02% in Q1 to 19.81% in Q4 as DI-GM quartiles increased. The Q4 group (highest DI-GM) has better socioeconomic and health behaviors: higher mean age (48.37 ± 15.53), PIR (3.43 ± 1.60), education level (>high school: 74.14%), and married/living with partner rate (66.32%). They also had the highest exercise rate (57.54%) and lowest smoking (43.67%) and drinking rates (13.53%) (*p* < 0.05). Additionally, non-Hispanic White participants had the highest representation in Q4 (73.36%), while the proportions of Mexican Americans and non-Hispanic Black participants decreased with increasing DI-GM (both *p* < 0.001).

**Table 1:** Baseline characteristics of the study population classified according to DI-GM.

Variable	Overall (weighted *N* = 160,008,955)	Q1 (*N* = 5,071)	Q2 (*N* = 5,454)	Q3 (*N* = 5,232)	Q4 (*N* = 5,343)	*p*-value
Age, years	45.81 ± 16.09	44.34 ± 16.23	44.73 ± 16.27	45.28 ± 16.08	48.37 ± 15.53	<0.001
PIR	3.08 ± 1.66	2.75 ± 1.64	2.98 ± 1.66	3.04 ± 1.66	3.43 ± 1.60	<0.001
Gender, *N* (weighted %)						<0.001
Male	11,081 (51.39)	2,873 (56.31)	2,896 (52.69)	2,710 (51.04)	2,602 (46.86)	
Female	10,019 (48.61)	2,198 (43.69)	2,558 (47.31)	2,522 (48.96)	2,741 (53.14)	
Race, *N* (weighted %)						<0.001
Mexican American	2,993 (8.37)	696 (8.60)	838 (9.84)	783 (8.62)	676 (6.58)	
Other Hispanic	2,121 (5.93)	536 (6.96)	563 (6.60)	521 (5.46)	501 (4.91)	
Non-Hispanic White	9,002 (67.42)	1924 (61.53)	2,255 (65.21)	2,277 (67.98)	2,546 (73.36)	
Non-Hispanic Black	4,758 (10.88)	1,461 (15.79)	1,257 (11.35)	1,166 (10.95)	874 (6.83)	
Other Race	2,226 (7.40)	454 (7.12)	541 (7.00)	485 (6.99)	746 (8.32)	
Levels of education, *N* (weighted %)						<0.001
≤ high school	9,232 (36.48)	2,630 (46.16)	2,590 (41.02)	2,269 (35.49)	1743 (25.86)	
> high school	11,868 (63.52)	2,441 (53.84)	2,864 (58.98)	2,963 (64.51)	3,600 (74.14)	
Marital status, *N* (weighted %)						<0.001
Never married	3,070 (14.97)	841 (16.40)	687 (13.42)	777 (17.85)	765 (13.11)	
Widowed/Divorced /Separated	5,489 (22.29)	1,360 (24.35)	1,591 (25.14)	1,318 (19.16)	1,220 (20.58)	
Married/Living with partner	12,541 (62.74)	2,870 (59.25)	3,176 (61.44)	3,137 (62.99)	3,358 (66.32)	
Drinking, *N* (weighted %)						0.002
Yes	3,591 (15.95)	981 (18.64)	961 (16.68)	882 (15.65)	767 (13.53)	
No	17,509 (84.05)	4,090 (81.36)	4,493 (83.32)	4,350 (84.35)	4,576 (86.47)	
Smoking, *N* (weighted %)						0.009
Yes	10,189 (46.42)	2,578 (48.70)	2,670 (47.17)	2,562 (46.86)	2,379 (43.67)	
No	10,911 (53.58)	2,493 (51.30)	2,784 (52.83)	2,670 (53.14)	2,964 (56.33)	
Exercise, *N* (weighted %)						<0.001
Yes	8,891 (48.44)	1826 (41.52)	2,167 (45.51)	2,196 (46.88)	2,702 (57.54)	
No	12,209 (51.56)	3,245 (58.48)	3,287 (54.49)	3,036 (53.12)	2,641 (42.46)	
MetS, *N* (weighted %)						<0.001
No	15,780 (78.24)	3,750 (75.98)	4,012 (76.55)	3,921 (79.87)	4,097 (80.19)	
Yes	5,320 (21.76)	1,321 (24.02)	1,442 (23.45)	1,311 (20.13)	1,246 (19.81)	

### The association between DI-GM and MetS

**Figure 2:**
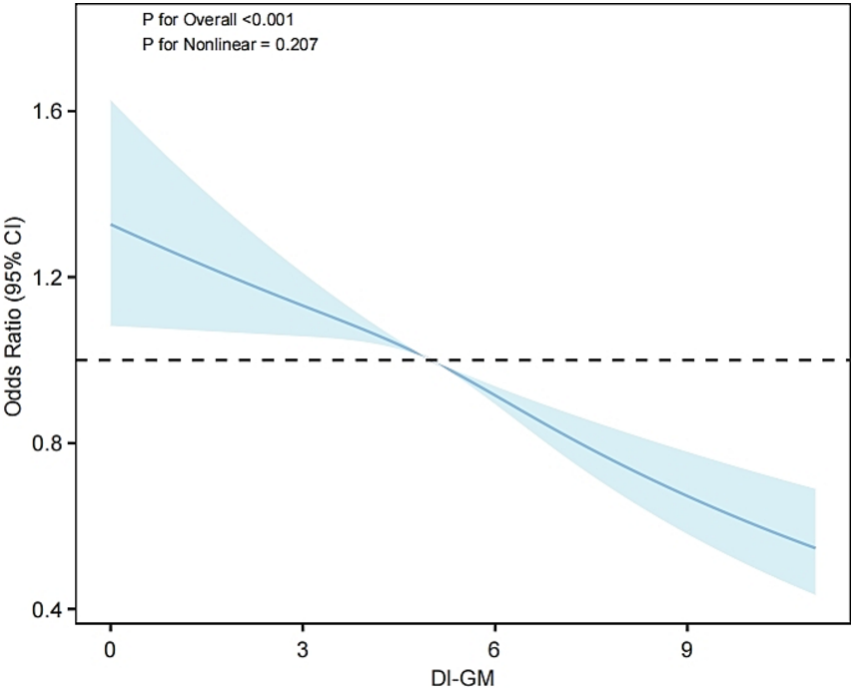
RCS analysis of the association between DI-GM and MetS.

Weighted multivariable logistic regression analysis was employed to evaluate the relationship between DI-GM and MetS (Table 2). In Model 1, with no adjustment for any covariates, a 1-unit increase in DI-GM was found to reduce MetS prevalence by 7% (OR = 0.93, 95% CI: 0.91–0.96, *p* < 0.001). Following further adjustment for age, sex, and race (Model 2), the reduction in prevalence increased to 10% (OR = 0.90, 95% CI: 0.87–0.93, *p* < 0.001). In the final model (Model 3), following additional adjustment for socioeconomic and lifestyle factors, each additional DI-GM unit was associated with an 8% reduction in MetS prevalence (OR = 0.92, 95% CI: 0.89–0.95, *p* < 0.001). The findings of the group analysis by DI-GM quartile demonstrated a significant 26% reduction in MetS prevalence in the highest quartile (Q4) in comparison to the lowest quartile (Q1) in Model 3 (OR = 0.74, 95% CI: 0.63–0.87, *p* < 0.001). Further analysis revealed that in Model 3, higher adverse scores for gut microbiota (i.e., reduced intake of harmful components) were associated with a lower prevalence of MetS (OR = 0.84, 95% CI: 0.80–0.88), with no significant differences observed for beneficial component scores. In addition, the beneficial ingredients avocados, chickpeas, coffee and the four ingredients unfavorable to gut microbes were all associated with a lower prevalence of MetS (*p* < 0.05) (see Supplementary Table S1). The trend analysis (*p* for trend < 0.001) additionally corroborated a statistically significant inverse relationship between DI-GM and metabolic syndrome prevalence. Furthermore, restricted cubic spline analysis (Figure 2) demonstrated a meaningful linear association between DI-GM and metabolic syndrome (*p* for overall < 0.001, *p* for nonlinear = 0.207), suggesting that elevated DI-GM scores correspond to reduced metabolic syndrome prevalence.

**Table 2:** Association between DI-GM and MetS.

Exposure	Model 1 OR (95% CI)	*p*-value	Model 2 OR (95% CI)	*p*-value	Model 3 OR (95% CI)	*p*-value
DI-GM	0.93 (0.91, 0.96)	<0.001	0.90 (0.87, 0.93)	<0.001	0.92 (0.89, 0.95)	<0.001
DI-GM quartiles						
Q1 (0–4)	Ref		Ref		Ref	
Q2 (4–5)	0.97 (0.83, 1.13)	0.689	0.95 (0.81, 1.11)	0.488	0.98 (0.83, 1.15)	0.773
Q3 (5–6)	0.80 (0.69, 0.93)	0.004	0.75 (0.64, 0.87)	<0.001	0.79 (0.68, 0.93)	0.004
Q4 (≥6)	0.78 (0.67, 0.91)	0.002	0.65 (0.56, 0.76)	<0.001	0.74 (0.63, 0.87)	<0.001
*p* for trend		<0.001		<0.001		<0.001
Beneficial to gut microbiota	0.97 (0.94,1.01)	0.139	0.95 (0.92,0.98)	0.005	0.99 (0.96,1.03)	0.598
Unfavorable to gut microbiota	0.88 (0.84,0.92)	<0.001	0.83 (0.80,0.87)	<0.001	0.84 (0.80,0.88)	<0.001

Model 1: No covariates were adjusted.

Model 2: Adjust for age, gender, race.

Model 3: Adjust age, gender, race, PIR, levels of education, Marital status, smoking, drinking, exercise.

### Subgroup analysis

**Figure 3:**
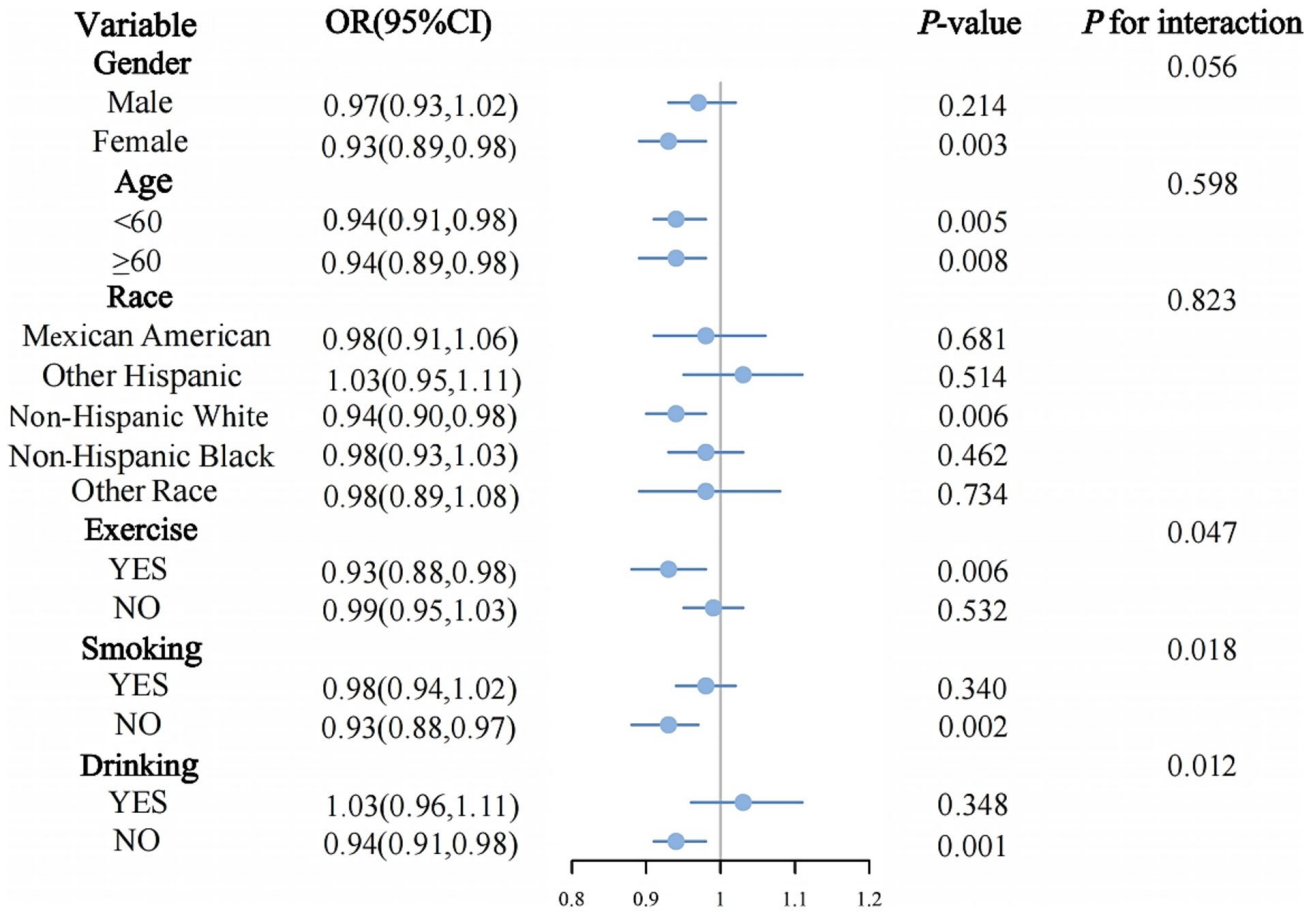
Subgroup analysis between DI-GM and MetS.

Subgroup analysis (Figure 3) demonstrated differing relationships between DI-GM and metabolic syndrome across various population segments. In exercise individuals (OR = 0.93, 95% CI: 0.88–0.98, *p* = 0.006), individuals who were non-smokers (OR = 0.93, 95% CI: 0.88–0.97, *p* = 0.002), and those who non-drinkers (OR = 0.94, 95% CI: 0.91–0.98, *p* = 0.001), the inverse relationship between DI-GM and metabolic syndrome reached statistical significance. Statistically significant interaction effects were detected for exercise (*p* for interaction = 0.047), smoking (*p* for interaction = 0.018), and drinking (*p* for interaction = 0.012). No significant interaction effects were observed in other subgroup analyses (all *p* for interaction > 0.05).

### Mediation analysis

**Figure 4:**
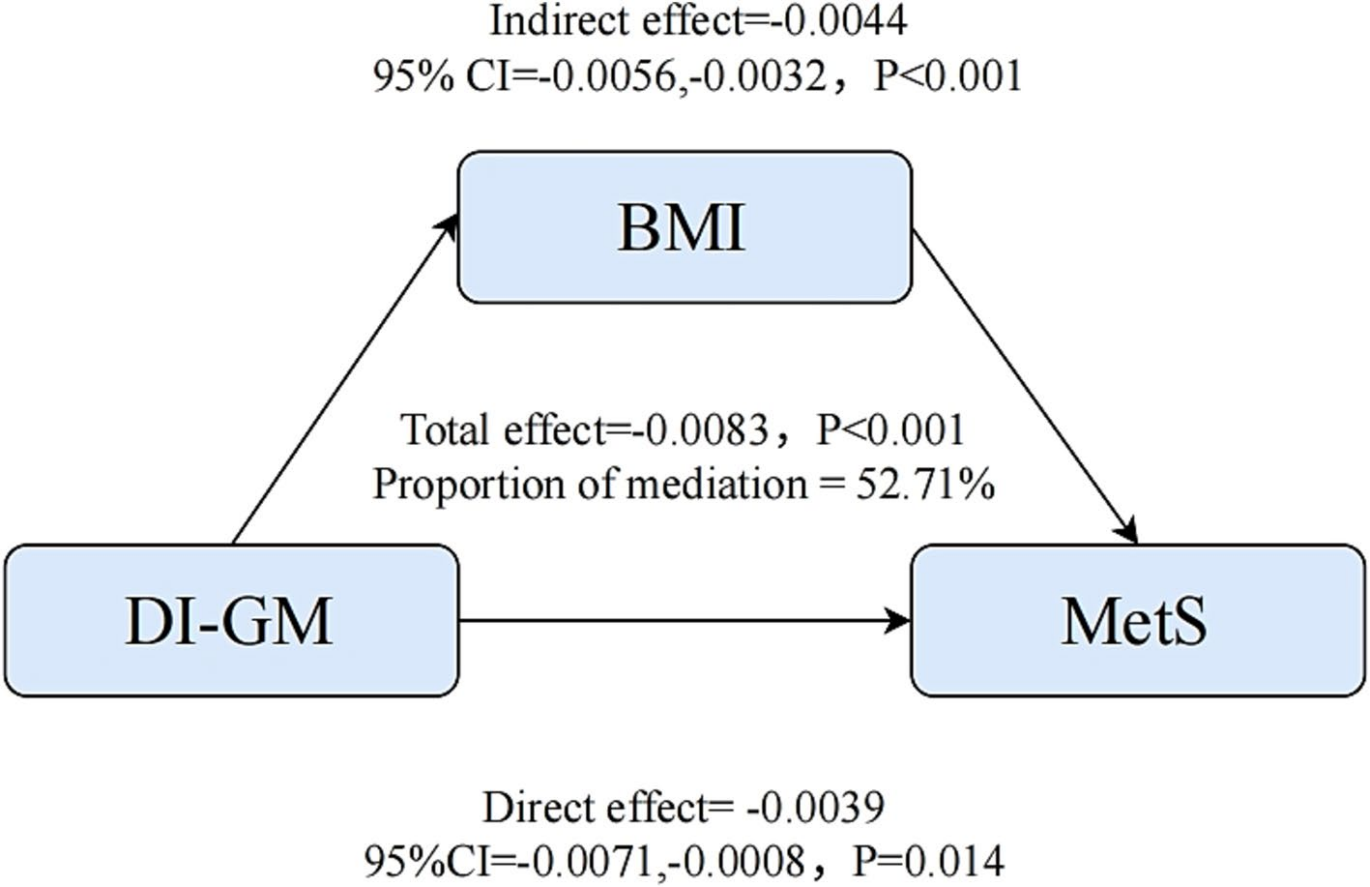
Mediation analysis.

Furthermore, mediation analysis was conducted to ascertain the potential mediating role of BMI in the association between DI-GM and MetS. As illustrated in Figure 4, the relationship between DI-GM and MetS was found to be significantly mediated by BMI, upon adjustment for all potential confounding variables. The total effect coefficient of DI-GM on BMI-mediated MetS was found to be −0.0083 (*p* < 0.001). The mediating effect was found to be −0.0044 (*p* < 0.001). The direct effect was found to be −0.0039 (*p* = 0.014). The proportion of mediation was 52.71% (*p* < 0.001).

## Discussion

This study analyzed the NHANES data from 2007 to 2020 to investigate the association between DI-GM and MetS. Findings revealed that higher DI-GM scores were linked to lower metabolic syndrome prevalence, even after adjusting for multiple covariates. RCS analysis additionally verified the linear association between these factors. Furthermore, this relationship was more evident among individuals maintaining healthy lifestyle behaviors, such as non-smoking, non-drinking, and exercising. It is important to note that BMI plays a key mediating role (mediating effect accounted for 52.71%). The findings indicate that dietary patterns targeting gut microbiota can reduce the risk of MetS by regulating body weight and directly improving metabolic homeostasis, thereby emphasizing the distinct value of DI-GM in comparison to conventional dietary indices.

It is important to emphasize that DI-GM is a proxy indicator constructed based on food components known to influence the composition and function of gut microbiota. Although DI-GM does reflect dietary patterns associated with gut health, it does not directly measure microbial diversity, composition, or metabolite levels. Consequently, the present findings indicate an association between diet and metabolic syndrome, which is mediated through putative microbial pathways rather than directly confirmed microbial causal changes. Notwithstanding this limitation, the dynamic interaction of dietary components with intestinal flora provides clear theoretical support for the underlying mechanism of DI-GM (see Supplementary Table S1 for details). Short-term dietary changes can quickly and reversibly alter microbiota composition, while long-term changes may lead to lasting changes in the microbial genome (13). DI-GM is designed on this principle by integrating 14 key dietary ingredients that affect gut flora, beneficial ingredients (e.g., whole grains, fermented dairy products, coffee) modulate insulin sensitivity by promoting short-chain fatty acid (SCFA) production, increasing the abundance of beneficial bacteria, improving intestinal barrier function, and inhibiting inflammation (14–16); harmful ingredients (e.g., red meat, high-fat diets) induce flora dysregulation, producing pro-inflammatory metabolites (e.g., TMAO), and exacerbating metabolic derangements (17–19). In comparison with conventional indices such as the Mediterranean Diet (MD) and the Dietary Approaches to Stop Hypertension (DASH), which demonstrate protective effects against MetS through anti-inflammation, modulation of lipid metabolism, or control of blood pressure (20, 21), the DI-GM is distinct in its approach. The DI-GM explicitly integrates foods that can be used as substrates for bacterial fermentation or that can inhibit bacterial dysbiosis (e.g., yogurt, kefir), and categorizes high-fat diets (≥40% of energy from fat) as unfavorable components (9). This dietary effect on flora is the result of multicomponent synergism intervening at the root of flora dysbiosis, providing a more mechanistic strategy for the prevention of MetS and a more precise tool for the assessment of diet-flora-metabolism interactions.

Further analysis in this study revealed that higher scores on the DI-GM score for harmful ingredients (i.e., lower intake of harmful ingredients) were associated with significantly lower prevalence of MetS. This finding is consistent with previous research that the DI-GM reduces the risk of diabetes by improving metabolic disorders (22). This suggests that reducing harmful ingredient intake is generalisable to improving the metabolic core mechanisms. However, scores on beneficial dietary components other than avocado, chickpeas, and coffee were not significantly associated with lower MetS prevalence. This phenomenon may be indicative of the heterogeneity of effects of different components in the diet-gut microbiota axis, or may be limited by the accuracy of short-term dietary assessments. Furthermore, it has been determined that specific beneficial components may exert a “double-edged sword” effect. In the male population, abnormal glucose metabolism has been identified as a significant contributor to the development of MetS, while the consumption of certain dairy products may indirectly lead to the onset of obesity and insulin resistance by increasing total calorie intake, a consequence of the presence of added sugars (23). Concurrently, excessive intake of green tea has the potential to attenuate its anticipated metabolic protective effects (24). The findings of this study suggest that a reduction in the consumption of detrimental substances (e.g., red meat, high-fat diets) may exert a more immediate effect on the prevention of MetS than merely increasing the consumption of beneficial substances.

Moreover, in subgroup analyses, the inverse correlation between DI-GM and MetS was more pronounced in exercise, non-smoking, and non-drinking individuals. However, it is important to note that these analyses were exploratory studies without statistical correction for multiple comparisons, which may increase the risk of false positives. Nevertheless, these results suggest a potential synergistic effect between DI-GM and health-promoting behaviors. These findings suggest a synergistic effect between DI-GM and health-promoting behaviors. Exercise improves gut microbiota composition and diversity by increasing beneficial bacteria, reducing gut inflammation, and enhancing intestinal barrier function (25). When combined with a high-quality DI-GM diet, they may synergistically enhance beneficial gut microbial effects, more effectively reducing MetS risk. Smoking and drinking are linked to dysbiosis and increased gut permeability (26, 27). Conversely, refraining from harmful habits maintains gut microbiota integrity, thereby enabling the beneficial components of DI-GM to more effectively regulate gut microbiota and support metabolic health. These interactions highlight the importance of combining dietary interventions with health-behavior promotion in MetS prevention and management, surpassing single-intervention limitations to maximize MetS risk reduction.

Notably, this study first quantified the mediating role of BMI in the association between the DI-GM score and MetS, expanding our understanding of the diet-gut microbiota-metabolism axis. These findings highlight weight management as a critical pathway through which diet improves metabolic health, likely due to the DI-GM diet’s high fiber content, abundant in whole grains and prebiotics such as fermented dairy products, that enhances satiety, reduces energy intake, and promotes BMI reduction (28). Additionally, restricting red meat and high-fat diets impedes fat accumulation, further alleviating visceral obesity (29). These results align with prior research showing that dietary interventions may improve metabolic disorders through the microbiota-metabolism axis and weight control (30). Importantly, the direct effect indicates the existence of BMI-independent pathways (e.g., enhancement of intestinal barrier), corroborating the “gut microbiota-metabolism” direct axis identified in earlier studies (31, 32). By verifying both the BMI-dependent and-independent mechanisms, this study enriches the theoretical framework for diet-metabolic disease relationships. Future research could deeply investigate the interaction between these two pathways using metabolomics, providing a more comprehensive understanding of dietary influences on metabolic health.

Although this study, based on NHANES 2007–2020 data, included 21,100 participants (representing 160 million after weighting), ensuring a large, nationally representative sample that enhances the universality and statistical power of the results. However, several limitations should be acknowledged. First, the cross-sectional design cannot establish a temporal relationship between DI-GM and MetS, raising the possibility of reverse causality. Individuals at high risk of MetS might alter their dietary behaviors due to metabolic abnormalities. Second, despite controlling for major confounders, unmeasured and unknown confounders could still influence the results. Third, dietary assessment relies on 24-h recalls, which may not accurately reflect long-term eating patterns and are susceptible to recall bias. Fourth, DI-GM infers gut microbiota status from dietary components but lacks direct biological data on microbiota composition, abundance, and functional metabolites. Lastly, NHANES samples are predominantly from the US, so extrapolating results to other ethnicities or cultural backgrounds should be done cautiously. In the future, it is recommended that the causality be verified through prospective cohorts and that the mechanism be explored in more depth using macrogenomic sequencing, in order to further verify whether the DI-GM-related dietary pattern plays a protective role through specific changes in the microbiota, and to carry out validation studies in multi-ethnic groups.

## Conclusion

This study demonstrated that DI-GM was significantly and negatively associated with the prevalence of metabolic syndrome (MetS), and that body mass index (BMI) played an important partial mediating role in this association. A healthy lifestyle (e.g., regular exercise, not smoking or drinking alcohol) could reinforce the protective effect of DI-GM. This suggests that dietary strategies targeting gut flora could directly regulate metabolic homeostasis through microbial metabolism and indirectly reduce the risk of MetS by managing weight. In the future, it will be necessary to analyze the molecular mechanism of the ‘diet-flora-BMI-metabolism’ axis by combining longitudinal cohort and multi-omics techniques. This will provide a basis for integrating dietary interventions and weight control strategies.

## Data Availability

Publicly available datasets were analyzed in this study. This data can be found here: all the datasets collected and analyzed during this study are available on the NHANES website (https://www.cdc.gov/nchs/nhanes/).
