# Supplementary material for: Association between the dietary index for gut microbiota and metabolic syndrome in adults: the mediating role of body mass index
Source: Front Nutr. 2025 Jul 16;12:1598664. doi: 10.3389/fnut.2025.1598664 (PMC12307214; doi:10.3389/fnut.2025.1598664)
Supplement: Supplementary file 1 [file Table_1.DOCX]

| Exposure | Model 1  OR (95%CI) | *p*-value | Model 2  OR (95%CI) | *p*-value | Model 3  OR (95%CI) | *p*-value |
| --- | --- | --- | --- | --- | --- | --- |
| **Beneficial to gut microbiota** |  |  |  |  |  |  |
| Avocados | 0.62(0.48, 0.79) | <0.001 | 0.64(0.50, 0.83) | 0.001 | 0.71(0.55, 0.92) | 0.012 |
| Broccoli | 0.89(0.75,1.04) | 0.132 | 0.86(0.72, 1.01) | 0.065 | 0.91(0.77, 1.08) | 0.289 |
| Chickpeas | 0.45(0.29, 0.71) | <0.001 | 0.48(0.30, 0.76) | 0.002 | 0.58(0.36, 0.92) | 0.022 |
| Coffee | 1.10(0.99, 1.21) | 0.071 | 0.87(0.77, 0.97) | 0.014 | 0.86(0.76, 0.98) | 0.020 |
| Cranberries | 0.91(0.77, 1.07) | 0.257 | 0.95(0.80, 1.13) | 0.591 | 0.94(0.80, 1.12) | 0.502 |
| Fermented dairy | 0.86(0.78, 0.94) | 0.002 | 1.06(0.96, 1.18) | 0.220 | 1.11(1.00, 1.22) | 0.045 |
| Fiber | 0.89(0.79,1.00) | 0.057 | 0.84(0.74,0.95) | 0.006 | 0.94(0.83,1.06) | 0.296 |
| Green tea | 1.39(1.23,1.58) | <0.001 | 1.37(1.20,1.56) | <0.001 | 1.37(1.21,1.56) | <0.001 |
| Soybean | 0.87(0.76,0.99) | 0.047 | 0.87(0.76,0.99) | 0.047 | 0.92(0.81,1.06) | 0.240 |
| Whole grains | 1.06(0.96,1.18) | 0.271 | 0.92(0.83,1.02) | 0.122 | 1.01(0.91,1.13) | 0.829 |
| **Unfavorable to gut microbiota** |  |  |  |  |  |  |
| Processed meat | 0.76(0.66,0.88) | <0.001 | 0.73(0.62,0.84) | <0.001 | 0.73(0.62,0.85) | <0.001 |
| Red meat | 0.82(0.74,0.90) | <0.001 | 0.76(0.69,0.84) | <0.001 | 0.79(0.71,0.87) | <0.001 |
| Fat | 0.78(0.69,0.88) | <0.001 | 0.84(0.74,0.95) | 0.005 | 0.83(0.74,0.94) | 0.005 |
| Refined grains | 1.03(0.93,1.16) | 0.548 | 0.83(0.74,0.93) | 0.002 | 0.83(0.73,0.93) | 0.002 |

**Supplementary Table S1** Association between the components of DI-GM and MetS

**Molecular mechanisms of gut microbiota and metabolic syndrome: dietary regulation and metabolic pathways**

**Association of gut microbiota dysbiosis with metabolic syndrome**

Dysfunctional composition and function of the gut microbiota is a key pathological hub connecting dietary patterns to Metabolic Syndrome (MetS). Several studies have found that obese individuals have reduced gut flora diversity and an increased thick-walled to anaplasmatoid phylum ratio. This change in flora structure may affect the host's energy metabolism and fat storage, thereby promoting obesity, one of the core components of MetS (1–3). Gut flora also affects metabolic homeostasis by producing various metabolites and inflammatory factors. Short-chain fatty acids (SCFAs) are the main metabolites produced by gut flora through the fermentation of dietary fibre and have various beneficial metabolic effects (4). For instance, short-chain fatty acids can regulate intestinal barrier function, reducing intestinal permeability and thus reducing systemic inflammatory responses triggered by bacterial products entering the bloodstream (5). At the same time, short-chain fatty acids can regulate pancreatic inflammatory responses by activating G protein-coupled receptors 41 (GPR41) and G protein-coupled receptors 43 (GPR43). This regulates insulin secretion and glucose metabolism and improves insulin resistance (6,7). Conversely, an imbalance in intestinal flora can lead to the overgrowth of harmful bacteria, resulting in endotoxemia and triggering a chronic inflammatory response. This facilitates the onset and development of insulin resistance and promotes the progression of MetS (8).

**Mechanisms of microbiota-metabolism axis modulation by DI-GM dietary components**

The beneficial ingredients in DI-GM, whole grains and legumes, are rich in insoluble fiber and dietary fiber, generate SCFA through microbial fermentation (9). The polyphenols in coffee and green tea promote the growth of beneficial bacteria and inhibit pathogens, acting as prebiotic substrates to increase SCFA (10). Fermented dairy products like yogurt and kefir are rich in probiotics. They introduce beneficial bacteria, enhance microbial diversity, and promote digestive system stability and gut health (11). These components increase beneficial bacterial abundance, boost SCFA production, strengthen gut barrier integrity, and reduce systemic inflammation, which are core mechanisms in insulin resistance and MetS development. Conversely, red meat and high-fat diets are adverse components of DI-GM. Intestinal microbiota processes carnitine and choline from red meat into trimethylamine (TMA), which hepatic flavin-containing monooxygenases further convert to trimethylamine-N-oxide (TMAO), a substance linked to atherosclerosis development (12,13). Diets high in fats lead to microbial imbalance, compromising gut barrier integrity and elevating circulating inflammatory markers and lipopolysaccharides (LPS), thereby initiating meta-inflammation and disturbing normal carbohydrate and lipid processing (14). These interconnected physiological changes exacerbate insulin resistance, weight gain, abnormal lipid levels, and elevated blood pressure, establishing a self-perpetuating cycle that heightens susceptibility to chronic metabolic disorders.

**References**

1. Aya V, Flórez A, Perez L, Ramírez JD. Association between physical activity and changes in intestinal microbiota composition: A systematic review. *PLoS One* (2021) 16:e0247039. doi: 10.1371/journal.pone.0247039

2. Li X, Li C. Analysis of changes in intestinal flora and intravascular inflammation and coronary heart disease in obese patients. *Exp Ther Med* (2018) 15:4538–4542. doi: 10.3892/etm.2018.5987

3. Mushraf S, Chawla K, Fayaz SMA, Mathew AJ, Reddy GPK, Kappettu Gadahad MR, Shenoy PA, Devi V, Adiga S, Nayak V. Exploring the effects of probiotics on olanzapine-induced metabolic syndrome through the gut microbiota. *Gut Pathog* (2024) 16:77. doi: 10.1186/s13099-024-00664-2

4. Carretta MD, Quiroga J, López R, Hidalgo MA, Burgos RA. Participation of Short-Chain Fatty Acids and Their Receptors in Gut Inflammation and Colon Cancer. *Front Physiol* (2021) 12:662739. doi: 10.3389/fphys.2021.662739

5. Li Z, Zhang F, Sun M, Liu J, Zhao L, Liu S, Li S, Wang B. The modulatory effects of gut microbes and metabolites on blood-brain barrier integrity and brain function in sepsis-associated encephalopathy. *PeerJ* (2023) 11:e15122. doi: 10.7717/peerj.15122

6. Łoniewski I, Szulińska M, Kaczmarczyk M, Podsiadło K, Styburski D, Skonieczna-Żydecka K, Bogdański P. Analysis of correlations between gut microbiota, stool short chain fatty acids, calprotectin and cardiometabolic risk factors in postmenopausal women with obesity: a cross-sectional study. *J Transl Med* (2022) 20:585. doi: 10.1186/s12967-022-03801-0

7. An Y, Dai H, Duan Y, Cheng L, Shi L, He C, Wang C, Lv Y, Li H, Zhang H, et al. The relationship between gut microbiota and susceptibility to type 2 diabetes mellitus in rats. *Chin Med* (2023) 18:49. doi: 10.1186/s13020-023-00717-9

8. Thomas MS, DiBella M, Blesso CN, Malysheva O, Caudill M, Sholola M, Cooperstone JL, Fernandez ML. Comparison between Egg Intake versus Choline Supplementation on Gut Microbiota and Plasma Carotenoids in Subjects with Metabolic Syndrome. *Nutrients* (2022) 14:1179. doi: 10.3390/nu14061179

9. Skeie G, Fadnes LT. Cereals and cereal products - a scoping review for Nordic Nutrition Recommendations 2023. *Food Nutr Res* (2024) 68: doi: 10.29219/fnr.v68.10457

10. Yang K, Jian S, Guo D, Wen C, Xin Z, Zhang L, Kuang T, Wen J, Yin Y, Deng B. Fecal microbiota and metabolomics revealed the effect of long-term consumption of gallic acid on canine lipid metabolism and gut health. *Food Chem X* (2022) 15:100377. doi: 10.1016/j.fochx.2022.100377

11. Kumar S, Mukherjee R, Gaur P, Leal É, Lyu X, Ahmad S, Puri P, Chang C-M, Raj VS, Pandey RP. Unveiling roles of beneficial gut bacteria and optimal diets for health. *Front Microbiol* (2025) 16:1527755. doi: 10.3389/fmicb.2025.1527755

12. Kolodziejczyk AA, Zheng D, Shibolet O, Elinav E. The role of the microbiome in NAFLD and NASH. *EMBO Mol Med* (2019) 11:e9302. doi: 10.15252/emmm.201809302

13. Curini L, Amedei A. Cardiovascular Diseases and Pharmacomicrobiomics: A Perspective on Possible Treatment Relevance. *Biomedicines* (2021) 9:1338. doi: 10.3390/biomedicines9101338

14. Li J, Li J, Ni J, Zhang C, Jia J, Wu G, Sun H, Wang S. Berberine Relieves Metabolic Syndrome in Mice by Inhibiting Liver Inflammation Caused by a High-Fat Diet and Potential Association With Gut Microbiota. *Front Microbiol* (2021) 12:752512. doi: 10.3389/fmicb.2021.752512
